# The *Euphausia superba* transcriptome database, SuperbaSE: An online, open resource for researchers

**DOI:** 10.1002/ece3.3168

**Published:** 2017-06-28

**Authors:** Benjamin J. Hunt, Özge Özkaya, Nathaniel J. Davies, Edward Gaten, Paul Seear, Charalambos P. Kyriacou, Geraint Tarling, Ezio Rosato

**Affiliations:** ^1^ Department of Genetics College of Medicine Biological Sciences and Psychology University of Leicester University Road Leicester UK; ^2^ British Antarctic Survey Natural Environment Research Council Cambridge UK

**Keywords:** Antarctic, circadian, crustacean, database, krill, transcriptome

## Abstract

Antarctic krill (*Euphausia superba)* is a crucial component of the Southern Ocean ecosystem, acting as the major link between primary production and higher trophic levels with an annual predator demand of up to 470 million tonnes. It also acts as an ecosystem engineer, affecting carbon sequestration and recycling iron and nitrogen, and has increasing importance as a commercial product in the aquaculture and health industries. Here we describe the creation of a *de novo* assembled head transcriptome for *E. superba*. As an example of its potential as a molecular resource, we relate its exploitation in identifying and characterizing numerous genes related to the circadian clock in *E. superba,* including the major components of the central feedback loop. We have made the transcriptome openly accessible for a wider audience of ecologists, molecular biologists, evolutionary geneticists, and others in a user‐friendly format at SuperbaSE, hosted at http://www.krill.le.ac.uk.

## 1 INTRODUCTION

Antarctic krill *Euphausia superba* (Dana, 1852; hereafter “krill”) is a keystone species of the Southern Ocean ecosystem, a hugely abundant pelagic crustacean inhabiting a circumpolar belt between the Antarctic continent and the Polar Front (Nicol, Constable, & Pauly, 2000), with an estimated total biomass of 379 million tonnes (Mt) and postlarval production of 342–536 Mt/yr (Atkinson, Siegel, Pakhomov, Jessopp, & Loeb, 2009). It sits at the center of a “wasp‐waist” food web (Atkinson et al., 2014) providing the major link between primary production and higher trophic levels and so converting phytoplankton to animal protein. Krill make up to 70% of the food intake of Southern Ocean predators such as seals, seabirds, whales, squid, and fish (Murphy et al., 2007), with an estimated annual demand of 128–470 Mt in the Southern Ocean ecosystem as a whole (Mori & Butterworth, 2006). Krill also have an impact on the abiotic environment as a suggested “ecosystem engineer” (Murphy et al., 2013), playing an important role in the biological pump through sequestering carbon from the ocean surface to ocean interior (Perissinotto, Gurney, & Pakhomov, 2000; Tarling & Johnson, 2006), as well as facilitating the recycling of iron (Schmidt et al., 2011) and the production and uptake of ammonium (Whitehouse, Atkinson, & Rees, 2011). Recent studies have suggested that krill may be vulnerable to habitat loss as an effect of warming, with particular impact in areas accessible by predators and used by fisheries (Hill, Phillips, & Atkinson, 2013), and through ocean acidification (Kawaguchi et al., 2013). Recruitment, which is dependent on the survival of krill larvae overwintering under sea ice, is considered particularly vulnerable to climate change (Flores et al., 2012), and it will be vital to understand and predict the adaptability of krill to a changing environment and the potential consequences as it does so.

Commercially, krill provide the region's largest fishery by weight at 300,000 tonnes annually, mostly used in aquaculture as a bulk ingredient or nutritional additive in fish meal (Nicol & Foster, 2016). In recent years, there has been notable growth in the demand for products for human use in the nutraceutical industry, exploiting the high omega‐3 polyunsaturated fatty acid content in krill. Krill oil is now the second most popular source of omega‐3 after fish oil (Backes & Howard, 2014). The oil is taken as a treatment or preventative measure for conditions such as cardiovascular disease and arthritis; 29% of recent krill‐related patents are categorized as for medical usage (Nicol & Foster, 2016).

As a keystone species of clear ecological and economic significance, active research is thriving, encompassing a broad spectrum of approaches from the population level down to the molecular. To cite further examples from recent years, this interest ranges from modeling population dynamics (Groeneveld et al., 2015) and food webs (Atkinson et al., 2012), through swarming behavior (Tarling et al., 2009), diurnal migratory behavior (Cresswell et al., 2009; Gaten, Tarling, Dowse, Kyriacou, & Rosato, 2008), and physiological stressor responses (Auerswald, Freier, Lopata, & Meyer, 2008) to peptide evolution and phylogeny (Cascella et al., 2015) and changes in gene expression related to seasonal or molt status (Seear, Goodall‐Copestake, Fleming, Rosato, & Tarling, 2012; Seear et al., 2010).

The enthusiasm for krill research is not always matched by the tractability of the organism itself, however; in contrast to the fruit fly *Drosophila melanogaster*, a model organism of some repute and power (Jennings, 2011), krill are remote, difficult to transport and maintain, and each discovery is hard won. The krill genome presents further challenges. Estimated at 47 gigabases (Jeffery, 2012), the genome of *E. superba* is more than twice the size of the largest genome sequenced so far, that of the loblolly pine *Pinus taeda* (Neale et al., 2014), and a sequencing project is considered unfeasible until further reductions in sequencing costs and improvements in long‐read technologies are achieved (Jarman & Deagle, 2016). Alternative approaches to genetic questions have therefore been adopted for krill, such as expressed sequence tag (EST) libraries (De Pittà et al., 2008; Seear et al., 2009), microarrays (Seear et al., 2010) and, with increasing popularity as the costs of RNA‐seq continue to fall, transcriptomic data that can provide gene coding sequences from reconstruction of mRNA transcripts (Clark et al., 2011; De Pittà et al., 2013; Martins et al., 2015; Meyer et al., 2015; Sales et al., 2017).

In this study, we describe the creation and annotation of SuperbaSE, a new de novo assembled head transcriptome for *E. superba* that is openly available online to interested researchers. With the aim of reconstructing as comprehensive a set of accurate, full‐length transcripts as possible, we used multiple variations of the de Bruijn graph method of *de novo* assembly, in which reads are broken down into a library of *k*‐mers, “words” of length *k*, prior to reassembly (Compeau, Pevzner, & Tesler, 2011). The output generated by this method varies with *k*‐mer length, with low values favoring transcript diversity and reconstruction of lowly expressed genes at the cost of fragmentation and accuracy, while high values favor higher accuracy over a broader abundance range, at the expense of diversity (Robertson et al., 2010). De Bruijn assembly software packages can also vary in their output when using the same *k*‐mer length (Xie et al., 2014).

Our own research interests revolve around the circadian clock, a field which until recently was lacking genetic data for crustaceans (Strauss & Dircksen, 2010), although matters have improved in recent years (Christie, Fontanilla, Nesbit, & Lenz, 2013; Tilden, McCoole, Harmon, Baer, & Christie, 2011; Zhang et al., 2013) and the head transcriptome was created as a gene discovery resource to drive progress in this area. Prior to the decision to adopt an RNA‐seq approach, we cloned and Sanger sequenced a number of core circadian genes for the krill through degenerate PCR and RACE extension; for some, we obtained complete coding sequences while achieving only partial success with others due to difficulties with degenerate PCR and RACE extension. As an example of the utility and potential of SuperbaSE, we relate here its use in completing our partial sequences and identifying many others relevant to the molecular clock, including regulatory genes involved in the generation and maintenance of circadian periodicity (Özkaya & Rosato, 2012) and those downstream whose expression is regulated by the clock (clock‐controlled genes).

## 2 MATERIALS AND METHODS

### 2.1 Animal collection

The collection of *E. superba* samples took place during the Antarctic summer in February 2008, on the Discovery 2010 cruise JR177. Swarms were identified using an EK60 echosounder North‐West of South Georgia at 52°S and caught by target fishing using a pelagic RMT8 net. Catches were taken at 1 a.m., 6 a.m., 1 p.m., and 8 p.m. local time. Immediately after collection between 30 and 200 individuals from each net were flash frozen by placing them into tubes that were then plunged into methanol at −80°C, moved to a −80°C freezer until the end of the cruise and returned to the UK without thawing.

### 2.2 RNA extraction

For each wild catch, three frozen krill heads were combined and powdered with mortar and pestle. Samples were kept frozen by continuous addition of liquid N_2_ and by working on a bed of dry ice. Total RNA was extracted with TRIzol^®^ reagent (Thermo Fisher Scientific, UK). RNA was resuspended in DEPC‐treated water and the concentration and purity [A(260 nm)/A(280 nm) >1.8] determined using a NanoDrop spectrophotometer (Thermo Fisher Scientific). One microgram of total RNA was electrophoresed on a nondenaturing 1.5% (w/v) agarose gel to check for degradation. The Qiagen oligotex^®^ mRNA extraction kit was used to isolate mRNA from one microgram of total RNA following the manufacturer's protocol. The mRNAs from each catch were combined into a single sample >10 μg and >250 ng/μl, which was delivered to BGI Tech Solutions (Hong Kong) Co Ltd (BGI) for generation of raw read data. The sample was subject to polyA enrichment using oligo‐dT beads, then fragmented and used to construct a cDNA library with an average fragment size of 200 base pairs (bp). This was primed with random hexamers and sequenced using the Illumina HiSeq 2000 platform to generate 100‐bp paired‐end reads.

### 2.3 *De novo* transcriptome assembly

The read data were subject to quality control by BGI, removing adapter sequences, contamination, and low‐quality reads, and once retrieved Fast QC v0.11.2 was used for further quality assessment and Trimmomatic 0.32 (Bolger, Lohse, & Usadel, 2014) to remove low quality leading and trailing bases.

Assembly was performed using Trinity r20140717 (Haas et al., 2013), Bridger r2014‐12‐01 (Chang et al., 2015), Trans‐ABySS 1.5.1 (Robertson et al., 2010), and SOAPdenovo‐Trans 1.03 (Xie et al., 2014). Trinity restricts the user to a *k*‐mer setting of 25. Bridger has a maximum permitted *k*‐mer of 31, and six assemblies were generated using *k*‐mers from 21 to 31 in two‐step increments. Trans‐ABySS and SOAPdenovo‐Trans can use higher *k*‐mer settings, and for each assembler, eight assemblies were created using *k*‐mers from 21 to 91 in 10‐step increments. A minimum contig length of 200 bp was specified for assembly.

For each assembler that permitted varying *k*‐mer settings, an assembler‐specific merged assembly was produced. For Trans‐ABySS, the built‐in function transabyss‐merge was used, while for the others the contig headers in each assembly were amended to avoid cross‐assembly duplicate IDs and the assemblies concatenated into a single FASTA file. These were then subject to the removal of redundancy using the dedupe.sh function of BBMap (http://sourceforge.net/projects/bbmap/), set to remove duplicates and containments at 100% identity including reverse complement comparisons.

Each merged assembly was assessed using the read metrics function of TransRate 1.01 (Smith‐Unna, Boursnell, Patro, Hibberd, & Kelly, 2016), which generates an output file of those contigs considered to be well assembled as supported by read‐mapping evidence. The “good contigs” files for each assembler were combined into a single file that was processed a second time using BBMap and TransRate to select the absolute best unique contigs across all assemblers. Going forward, this selection of contigs is referred to as the total assembly.

Transdecoder (Haas et al., 2013) was used to identify likely coding contigs with an open reading frame coding for peptides of 100 amino acids (aa) or longer—only the longest identified ORF per contig was retained. The output was processed using CD‐HIT (Fu, Niu, Zhu, Wu, & Li, 2012) at 100% identity to remove duplicates, resulting in the peptide assembly. The contigs from the total assembly that encode these peptides were extracted into a new database, hereafter referred to as the coding assembly.

### 2.4 Transcriptome annotation and analysis

The peptide assembly was queried against the SwissProt protein database from the UniProt Knowledgebase (UniProtKB) using the blastp function of BLAST+ 2.5.0 (Camacho et al., 2009). The output was set to return the single best result (‐max_target_seqs 1, ‐max_hsps 1) with an E‐value of 1.0e^−6^ or lower. All contigs from the peptide assembly that failed to return a result were subsequently queried against the arthropod protein database of the UniProtKB with the same criteria. Both protein databases were retrieved from ftp://ftp.ebi.ac.uk/pub/databases/fastafiles/uniprot/ on 26th November 2016. The coding assembly was also subject to sequence homology annotation using the blastx function of BLAST+ against the two protein databases using the same method. The accessions returned by these searches were used to retrieve associated Enzyme Codes, GO terms, KEGG, and InterProScan IDs from the UniProt website (http://www.uniprot.org/uploadlists/) using the Retrieve/ID mapping function. The peptide assembly was further annotated using functions built into Trinotate (Haas et al., 2013) to identify Pfam protein domains.

To provide an overview of the information present in our assembly and compare it to the current most extensive krill molecular resource, KrillDB (Sales et al., 2017), we used blastx annotation results from the high quality, manually reviewed SwissProt database as a metric. The SuperbaSE coding assembly, KrillDB (retrieved from http://krilldb.bio.unipd.it/ on 14 March 2017) and the mRNA transcripts derived from the genome of another crustacean, *Daphnia pulex* (Colbourne et al., 2011; retrieved from http://wfleabase.org/genome/Daphnia_pulex/current/mrna on 5th April 2017) were subject to this search using an E‐value cutoff of 1.0e^−6^ or lower, the output again set to return the single best result. Results were used to retrieve GO annotation from UniProtKB as above and summarized using WEGO (Ye et al., 2006). To identify the extent of the overlap between KrillDB and the coding assembly, the two resources were further subject to a reciprocal BLAST search using blastn, E‐value 1.0e^−20^ or lower, returning the single best result.

### 2.5 SuperbaSE: the online *Euphausia superba* transcriptome database

The transcript and annotation data of the peptide and coding assemblies were merged, processed and converted into an appropriate format for online viewing. Each entry was given sequential and consistently formatted gene, transcript and ORF IDs and assigned putative gene names based on BLAST annotation results. The site was created using a Catalyst framework to access annotated transcriptome data stored in a MySQL database, and the front‐end design makes use of template toolkit and Twitter Bootstrap.

### 2.6 Transcriptome mining

Coding sequences for *E. superba bmal1* (*Es‐bmal1*, GenBank accession KX238952)*, clock* (*Es‐clk*, KX238953), *cryptochrome 1* (*Es‐cry1*, KX238951), and *cryptochrome 2* (*Es‐cry2*, also reported as *Escry* by Mazzotta et al. (2010)) had already been successfully cloned and Sanger sequenced in full when the head transcriptome project was undertaken, while *E. superba period* (*Es‐per,* KX238955) and *timeless* (*Es‐tim,* KX238954) had both been partially cloned and sequenced (Table 1 and Supplementary Data). These sequences were used to query the total assembly to determine the extent and accuracy of their reconstruction in the transcriptome and, in the case of *Es‐per* and *Es‐tim*, to obtain the full coding sequences. The queries were performed by translation of the nucleotide sequences to putative peptides using ExPASy Translate (http://web.expasy.org/translate/), with these then used to mine the total assembly using the tblastn function of BLAST+ with an E‐value cutoff of 1.0e^−3^. For *Es‐per* and *Es‐tim*, the output was used to design primers for confirmation of the full coding sequences of both genes as outlined above (Supplementary Data, Table S2).

**Table 1:** Core circadian genes cloned and Sanger sequenced for *Euphausia superba. Es‐cryptochrome 2* has been previously reported as *Escry* (Mazzotta et al., 2010)

Gene name	Accession	Length (nt/aa)			Top NCBI nr hit				
		mRNA	CDS	Peptide	Accession	Name	Species	*E*‐value	Identity (%)
*Es‐bmal1*	KX238952	2,017	1,992	664	AFV39705	*bmal1a*	*Pacifastacus leniusculus*	0	73
*Es‐clock*	KX238953	4,044	4,032	1,344	AAX44045	*clock*	*Macrobrachium rosenbergii*	0	79
*Es‐cryptochrome 1*	KX238951	1,644	1,599	533	AJY53623	*cryptochrome 1*	*Nilaparvata lugens*	0	53
*Es‐cryptochrome 2*	*–*	1,720	1,635	545	CAQ86665	*cryptochrome*	*Euphausia superba*	0	100
*Es‐period*	KX238955	4,061	3,783	1,261	ALC74274	*period‐like protein*	*Nephrops norvegicus*	0	67
*Es‐timeless*	KX238954	4,222	3,933	1,311	ALC74275	*timeless‐like protein*	*Nephrops norvegicus*	0	75

For regulatory and clock‐controlled genes, the peptide sequences of *D. melanogaster/Mus musculus* genes known to contribute to or interact with circadian timekeeping were used to search the total assembly for putative *E. superba* orthologs using the tblastn function of BLAST+ with an E‐value cutoff of 1.0e^−3^. To add additional support to such identification, candidate contigs were extracted from the assembly database and translated, and the peptide sequences blasted against *D. melanogaster* proteins curated in Flybase (Attrill et al., 2016) and against the NCBI nonredundant proteins database (excluding uncultured/environmental sample sequences and all entries with a title containing the words “putative,” “hypothetical,” or “predicted”). The same approach was taken in identifying a selection of neuropeptides (derived from Gard, Lenz, Shaw, & Christie, 2009; Ma et al., 2009; Christie, Durkin, Hartline, Ohno, & Lenz, 2010; Christie, Stemmler, & Dickinson, 2010; Ma et al., 2010; Christie, McCoole, Harmon, Baer, & Lenz, 2011; Christie, Nolan, Ohno, Hartline, & Lenz, 2011; Christie, Roncalli, et al., 2013) that might potentially be under the control of the biological clock in *E. superba*.

The derived peptide sequences obtained from this process were used to search KrillDB with the tblastn function with an E‐value cutoff of 1.0e^−3^ to compare the presence and extent of transcript reconstruction of circadian‐related genes in the two resources. In each case, KrillDB was also queried with the *D.melanogaster/M.musculus* ortholog used in the initial search process to search this resource for superior candidates.

## 3 RESULTS

### 3.1 Sequencing data and quality control

34,918,657 clean paired‐end 100‐bp reads (69,837,314 in total) were generated by the Illumina sequencing. After Trimmomatic processing for low‐quality bases at each end of all reads, the read lengths ranged between 81 and 100 bases.

### 3.2 Assembly and annotation metrics

The total assembly initially comprised 484,125 contigs. Submission to the NCBI Transcriptome Shotgun Assembly database required the assembly to be subject to a contamination screen, with 45 contigs removed as a result and 484,080 contigs submitted to the TSA under accession GFCS00000000. The total assembly achieved a TransRate score of 0.42, an improvement over all the individual multi‐ or single *k*‐mer assemblies produced by any one assembly package (Table 2). There were also improvements for the total assembly in the percentage of total read mappings, the percentage of good read mappings, the percentage of contigs with identified open reading frames (ORFs), and a reduction in the number of perceived bridges, which indicate transcript fragmentation. The contig mean length and assembly N50 of the total assembly were notably higher than the output of Trinity, SOAPdenovo‐Trans, and Trans‐ABySS, yet lower than the output of Bridger, signaling a proportionally large contribution of contigs from the latter; indeed, Bridger provided 50% of contigs in the total assembly, followed by Trans‐ABySS, SOAPdenovo‐Trans, and Trinity with 26%, 15%, and 9% respectively. From the total assembly, TransDecoder identified 147,450 contigs (the coding assembly) encoding putative peptides (the peptide assembly). Of these, 27,413 were deemed by TransDecoder to represent complete peptides; 60,826 were identified as internal fragments, 42,311 as missing the 5′ end and 16,900 as missing the 3′ end.

**Table 2:** Comparison of *de novo* assembly quality using TransRate contig and read‐mapping metrics. Bridger, SOAPdenovo‐Trans, and Trans‐ABySS assemblies are multi‐*k*‐mer assemblies, see Methods for details. See Smith‐Unna et al. (2016) and http://hibberdlab.com/transrate/metrics.html for further details regarding TransRate metrics. Read‐mapping metrics were not considered appropriate for the coding assembly, which is a selective dataset

Metric	Bridger	SOAPdenovo‐Trans	Trans‐ABySS	Trinity	Total assembly	Coding assembly
No. of contigs	513,499	282,833	318,436	157,274	484,080	147,450
Shortest	189	200	200	201	200	297
Longest	27,543	17,265	37,689	11,148	34,468	34,468
Mean length	780	445	573	503	726	1,121
No. with ORF	138,937	51,336	72,597	31,566	135,969	100,823
ORFs %	27%	18%	23%	20%	28%	81%
N50	1,218	480	738	602	1,071	1,518
% of reads mapped	97%	93%	92%	82%	98%	–
Good mappings	30,880,238	28,292,720	28,688,483	22,012,681	31,833,986	–
% good mappings	88%	81%	82%	63%	91%	–
Potential bridges	43,048	46,708	38,398	38,011	34,120	–
% uncovered bases	38%	11%	15%	4%	23%	–
% contigs with uncovered bases	86%	71%	77%	44%	80%	–
% contigs uncovered	45%	16%	18%	4%	29%	–
% contigs low‐covered	93%	75%	80%	76%	89%	–
Transrate score	0.13	0.26	0.25	0.21	0.42	–

BLAST annotation of the peptide assembly by sequence homology returned 67,762 peptide contigs with hits from the UniprotKB SwissProt dataset, with an additional 20,972 contigs annotated using the UniprotKB arthropod dataset. A further 3,467 contigs were annotated by querying SwissProt and the arthropod dataset with the coding assembly nucleotide sequences for an overall total of 92,201 contigs annotated in either peptide or nucleotide form. Of these contigs, 80,149 were subsequently annotated with at least one GO term, 57,094 with at least one KEGG identifier, and 86,405 with at least one InterProScan accession. Further to this, 67,782 contigs were annotated with Pfam domain identifiers (whole sequence E‐value of 1.0e^−6^ or lower), including 2,044 contigs that were not BLAST annotated.

The number of unique accessions returned by this BLAST annotation process was 23,931; of these, 10,762 were assigned to a single contig, while the rest were assigned to two contigs or more to reach the total number. The SwissProt entry Q9V7U0, for example, a pro‐resilin precursor from *Drosophila melanogaster,* annotated 1,148 contigs, and another 32 unique accessions were each assigned to 100 contigs or more. By way of comparison, a single assembly generated using Bridger at *k*‐mer = 25 (Bridger25) received 12,616 unique accessions from SwissProt (Supplementary Data, Table S3). Q9V7U0 was assigned to 274 contigs in this assembly, suggesting a duplication factor of 3.8 in the total assembly; of the SwissProt accessions assigned to 100 contigs or more in the peptide and coding assemblies, the duplication factor varied from 2.03 to 8.2.

**Figure 1:**
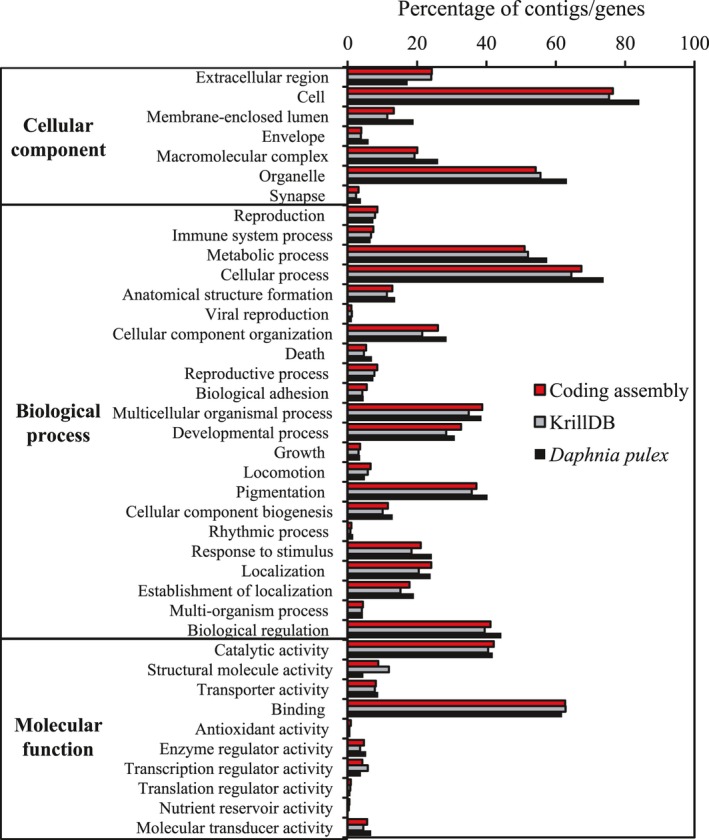
Functional characterization of SuperbaSE coding assembly, in comparison with KrillDB (also *E. superba*) and *D. pulex* mRNA transcripts. Chart depicts percentage of contigs/genes mapping to high‐level GO terms retrieved through BLAST annotation with SwissProt

Figure 1 shows the high‐level summary of the functional annotation of the coding assembly, KrillDB and the *Daphnia pulex* transcripts using SwissProt/GO mapping. The content of the two krill resources is highly similar at this level, and there appear to be no major functional categories missing or underpresented in comparison with the genome‐derived transcripts of *D. pulex*. Comparing the underlying SwissProt annotation, results showed that 67,206 coding assembly contigs were annotated by 15,441 unique accessions, 7,492 (48.5%) of which were not seen in the KrillDB results. KrillDB saw 65,248 annotated contigs in total with 10,887 unique accessions, 2,938 (27%) of which were not seen in the coding assembly results. In the results of the reciprocal BLAST, no match was found in KrillDB for 34,768 contigs from the coding assembly (23.6%), while 19,270 contigs from KrillDB had no match in the coding assembly (14.4%).

### 3.3 Transcriptome mining

**Figure 2:**
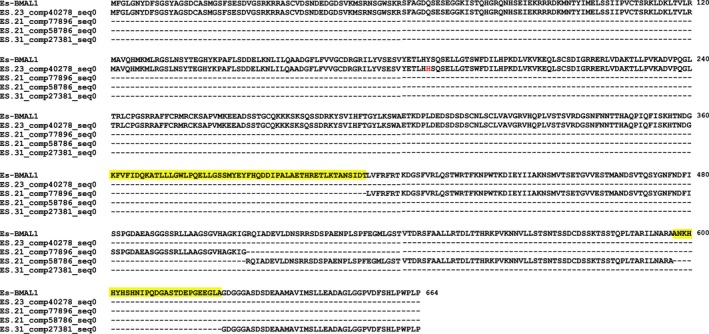
Es‐BMAL1 (accession KX238952) aligned with fragments mined from the total assembly. Yellow highlights represent sequence data not found in the total assembly

**Figure 3:**
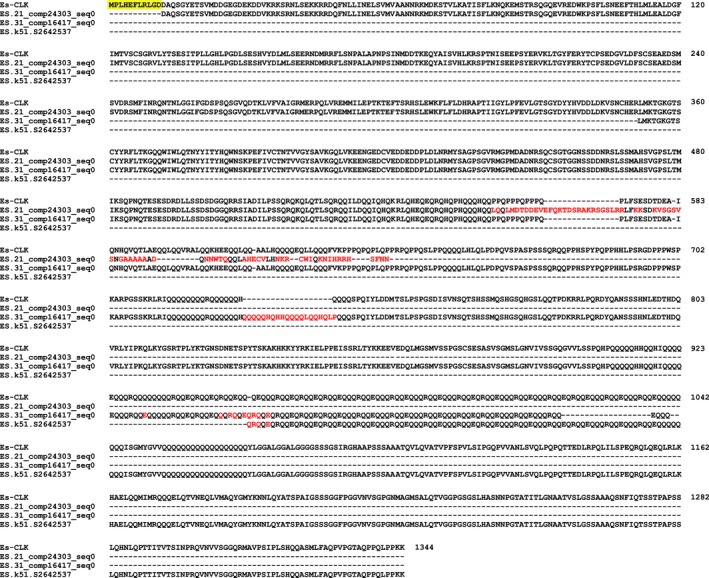
Es‐CLOCK (accession KX238953) aligned with fragments mined from the total assembly. Yellow highlights represent sequence data not found in the total assembly. Red text highlights residues inconsistent between the confirmed PCR sequence and transcriptome sequences

**Figure 4:**
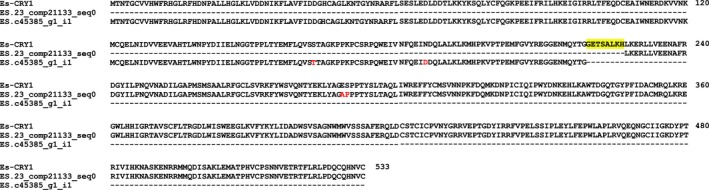
Es‐CRY1 (accession KX238951) aligned with fragments mined from the total assembly. Yellow highlights represent sequence data not found in the total assembly. Red text highlights residues inconsistent between the confirmed PCR sequence and transcriptome sequences

**Figure 5:**
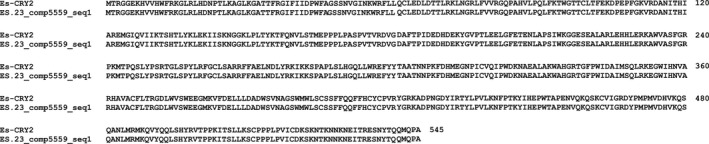
Es‐CRY2 (accession CAQ86665, Mazzotta et al. (2010)) aligned with contig mined from the total assembly

**Figure 6:**
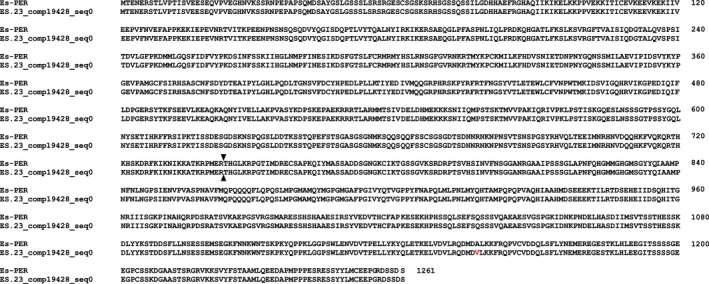
Es‐PERIOD (accession KX238955) aligned with contig mined from the total assembly. Red text highlights residues inconsistent between the confirmed PCR sequence and transcriptome sequence. Black arrows denote the point at which sequence data from 3′ RACE extension ends—beyond this the transcriptome was necessary to complete the sequence

**Figure 7:**
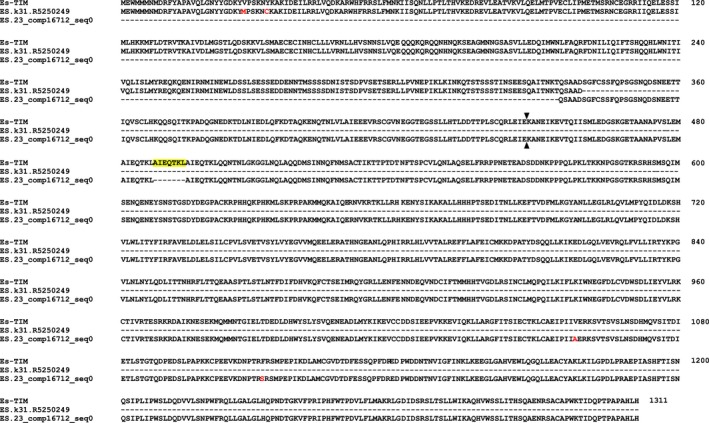
Es‐TIMELESS (accession KX238954) aligned with contig mined from the total assembly. Yellow highlights represent sequence data not found in the total assembly. Red text highlights residues inconsistent between the confirmed PCR sequence and transcriptome sequence. Black arrows denote the point at which sequence data from 3′ RACE extension ends—beyond this the transcriptome was necessary to complete the sequence

Searching the total assembly using the complete coding sequences of *Es‐bmal1* (Figure 2), *Es‐clk* (Figure 3), *Es‐cry1* (Figure 4), and *Escry2* (Figure 5) returned transcripts representing between 88 and 100% of each sequence; in the case of *Es‐cry2,* this was a single, complete transcript, while the others returned 2–4 fragments. Searching the assembly using the fragments of *Es‐per* and *Es‐tim* returned a complete coding sequence for the former (Figure 6), and two large fragments, covering nearly the complete sequence, for the latter (Figure 7). In both cases, these full sequences were subsequently confirmed by PCR, cloned, and Sanger sequenced (Supplementary Data and Table 1). In some cases, minor variations were seen when comparing the peptide sequences derived from assembled transcripts to those confirmed by high fidelity PCR, and in the case of Es‐CLK, there is a notable stretch of disagreement with particular contigs from residues 561–641, and a smaller stretch later on (Figure 3).

Table 3 shows the putative regulatory and clock‐controlled genes mined from the transcriptome, while Table 4 shows the results of BLAST searches against Flybase and NCBI nonredundant (NR) peptide databases using these output contigs to support their assigned identities. With the exception of *jetlag,* a putative ortholog was identified for all the 22 genes we attempted to identify, 18 of which were represented by full coding sequences. *E. superba* pigment‐dispersing hormone (PDH) has been previously reported (Toullec et al., 2013) as Eus‐PDH β, and the contig identified as *Es‐pdh1* (ES.k51.S2746663) is in agreement with this. Table 5 shows the neuropeptides similarly identified and characterized using only the NCBI NR database; of the 31 preprohormones used as queries 21 returned results, all of which were accepted as putative *E. superba* orthologs. Candidate peptide sequences for *E. superba* allatostatin C, bursicon α, corazonin, and neuroparsin have previously been reported by Toullec et al. (2013), although none are in perfect agreement with our contigs.

**Table 3:** Transcriptome mining: clock‐related query proteins and *E. superba* output contigs

Query Protein	Accession	*E. superba* transcript/protein identifications		Length	Protein name	Length
Protein name		Contig ID	*E*‐value			
CASEIN KINASE II α	AAN11415	ES.21_comp3643_seq1	0	2556	Es‐CKIIα	350
CASEIN KINASE II β	AAF48093	ES.194593	4E‐119	1276	Es‐CKIIβ	219
CLOCKWORK ORANGE	AAF54527	ES.21_comp15386_seq1	2E‐26	2301	Es‐CWO	707
CTRIP	AAF52092	ES.31_comp9752_seq0	1E‐167	8148	Es‐CTRIP	2152
DOUBLETIME	AAF57110	ES.25_comp4349_seq1	0	2480	Es‐DBT	345
JETLAG	AAF52178	ES.21_comp16370_seq0	1E‐15	1901	–	–
LARK	AAF50578	ES.k31.S5232406	6E‐59	1199	Es‐LARK	326
NEJIRE	AAF46516	ES.31_comp5882_seq0	0	6924	Es‐NEJ	2072^c^
NEMO	AAF50497	k21.S7956922	0	2038	Es‐NEMO	456^c^
PAR DOMAIN PROTEIN 1ε	AAF04509	ES.k41.J3781389	8E‐05	1835	Es‐PDP1	489
PIGMENT DISPERSING FACTOR^a^	AAF56593	ES.k51.S2746663	8E‐06	387	Es‐PDH1	74
		ES.c68106_g1_i1	3E‐05	531	Es‐PDH2	79
PIGMENT DISPERSING FACTOR RECEPTOR	AAF45788	ES.21_comp12084_seq0	3E‐91	1588	Es‐PDHR	295
PROTEIN PHOSPHATASE 1	CAA39820	ES.k51.J2636217	0	1870	Es‐PP1	329
PROTEIN PHOSPHATASE 2A ‐ MICROTUBULE STAR	AAF52567	ES.23_comp1445_seq0	0	2263	Es‐MTS	309
PROTEIN PHOSPHATASE 2A ‐ TWINS	AAF54498	ES.21_comp997_seq2	0	3375	Es‐TWS	455
PROTEIN PHOSPHATASE 2A ‐ WIDERBORST	AAF56720	ES.104836	0	4190	Es‐WBT	461
REV‐ERBα^b^	NP_663409	ES.25_comp2884_seq0	1E‐45	3602	Es‐E75	800
SHAGGY	AAN09084	ES.27_comp3209_seq0	0	2222	Es‐SGG	415
SUPERNUMERARY LIMBS	AAF55853	ES.25_comp34876_seq0	0	1843	Es‐SLIMB	612^c^
TAKEOUT	AAF56425	ES.21_comp3588_seq0	1E‐11	1090	Es‐TAKEOUT	247
TIMELESS (TIMEOUT)^b^	Q9R1X4	ES.23_comp37221_seq0	2E‐82	934	Es‐TIMEOUT	1363^d^
		ES.29_comp20079_seq0	4E‐94	3067		
VRILLE	AAF52237	ES.25_comp4277_seq0	3E‐43	2021	Es‐VRI	474

a *E. superba* PDH also reported by Toullec et al. (2013).

b Query protein used is a *Mus musculus* orthlog. All others from *Drosophila melanogaster*.

c Es‐NEJIRE, Es‐NEMO and Es‐SLIMB are 3′ partial fragments.

d Es‐TIMEOUT full protein sequence determined through PCR using fragments listed.

**Table 4:** Results of blastp analyses of putative *E. superba* clock‐associated proteins against *D. melanogaster* protein database (Flybase) and NCBI nonredundant (nr) protein database

Query	Top Flybase hit			Top NCBI nr hit			
	Flybase No.	Associated gene name	*E*‐value	Accession	Name	Species	*E*‐value
Es‐CKIIα	FBpp0070041	*casein kinase IIa*	1E‐162	EFN84867	Casein kinase II subunit alpha	*Harpegnathos saltator*	0
Es‐CKIIβ	FBpp0300427	*Casein kinase II ß*	6E‐114	ELR50200	Casein kinase II subunit beta	*Bos mutus*	5E‐146
Es‐CTRIP	FBpp0310477	*circadian trip*	1E‐173	ACC99349	ULF (TRIP12)	*Homo sapiens*	0
Es‐CWO	FBpp0081723	*clockwork orange*	8E‐27	KDR16323	Hairy/enhancer‐of‐split related with YRPW motif protein 2	*Zootermopsis nevadensis*	2E‐61
Es‐DBT	FBpp0306615	*discs overgrown*	7E‐157	AGV28719	Casein kinase 1 epsilon	*Eurydice pulchra*	0
Es‐E75	FBpp0074915	*Ecdysone‐induced protein 75B*	2E‐143	AGS94407	Ecdysteroid receptor E75	*Litopenaeus vannamei*	0
Es‐LARK	FBpp0076555	*lark*	2E‐55	NP_523957	lark, isoform A	*Drosophila melanogaster*	2E‐62
Es‐NEJ^a^	FBpp0305701	*nej*	0	KDR19833	CREB‐binding protein	*Zootermopsis nevadensis*	0
Es‐NEMO^a^	FBpp0076474	*nemo*	0	NP_729316	Nemo, isoform E	*Drosophila melanogaster*	0
Es‐PDP1	FBpp0076495	*PAR‐domain protein 1*	2E‐35	EFN83234	Hepatic leukemia factor	*Harpegnathos saltator*	2E‐39
Es‐PDH1	FBpp0084396	*Pigment‐dispersing factor*	3E‐05	JC4756	PDH related peptide precursor 79	*Penaeus sp*.	9E‐27
Es‐PDHR	FBpp0309084	*Pigment‐dispersing factor receptor*	9E‐97	BAO01102	Neuropeptide GPCR B2	*Nilaparvata lugens*	1E‐140
Es‐PP1	FBpp0306442	*Protein phosphatase 1a at 96A*	4E‐178	EKC23784	PP1‐alpha catalytic subunit	*Crassostrea gigas*	0
Es‐MTS	FBpp0310063	*microtubule star*	2E‐176	KDR18186	PP2A catalytic subunit alpha	*Zootermopsis nevadensis*	0
Es‐TWS	FBpp0081671	*twins*	0	AFK24473	PP2A regulatory subunit B	*Scylla paramamosain*	0
Es‐WBT	FBpp0084575	*widerborst*	0	XP_002427654	PP2A regulatory subunit epsilon	*Pediculus humanus corporis*	0
Es‐TAKEOUT	FBpp0297106	*CG2016*	1E‐32	ACO12182	Circadian clock‐controlled protein precursor	*Lepeophtheirus salmonis*	1.00E‐47
Es‐TIMEOUT	FBpp0082180	*timeout*	0	EEZ99220	Timeout	*Tribolium castaneum*	0
Es‐SGG	FBpp0070450	*shaggy*	0	AEO44887	Shaggy	*Tribolium castaneum*	0
Es‐SLIMB^a^	FBpp0306059	*supernumerary limbs*	0	KDR19729	F‐box/WD repeat‐containing protein 1A	*Zootermopsis nevadensis*	0
Es‐VRI	FBpp0309715	*vrille*	4E‐42	AAT86041	Vrille	*Danaus plexippus*	3E‐68

a Fragment. Es‐PDH2 is not shown as it is a minor variant of Es‐PDH1.

**Table 5:** Preprohormone query proteins with *E. superba* output contigs and subsequent blastp analysis against NCBI nonredundant protein database

Peptide family (subfamily)	Output from total assembly using Accession as query				Output from NCBI NR using total assembly contig as query		
	Accession	Contig	Size (nt)	*E*‐value	Accession	Description	*E*‐value
Allatostatin A	ABS29318	ES.27_comp22909_seq0	1239	4E‐19	BAF64528	allatostatin precursor protein *Panulirus interruptus*	1E‐60
Allatostatin B	AAF49354	ES.27_comp14162_seq0	710	2E‐09	AFV91538	B‐type preproallatostatin I *Pandalopsis japonica*	3E‐15
Allatostatin C^a^	AAF53063	ES.23_comp57987_seq0	757	4E‐05	BAO00935	Allatostatin‐cc *Nilaparvata lugens*	5E‐13
Allatotropin	AAB08757	–	–	–	–	–	–
Bursicon alpha^a^	EFX87546	ES.21_comp33157_seq0	552	2E‐60	AKJ74864	Bursicon alpha subunit *Penaeus monodon*	4E‐72
Bursicon beta	EFX87749	ES.21_comp28222_seq0	721	7E‐37	AKJ74865	Bursicon beta subunit *Penaeus monodon*	9E‐63
CHHamide	NP_001097784	ES.25_comp21895_seq1	829	6E‐04	EFX80320	CCHamide‐like precursor *Daphnia pulex*	3E‐08
Corazonin^a^	AAB32283	ES.29_comp12038_seq1	365	2E‐04	Q9GSA4	Corazonin precursor‐related peptide *Galleria mellonella*	2E‐06
Crustacean cardioactive peptide	EFX70015	ES.27_comp31900_seq0	685	2E‐02	ABB46291	Crustacean cardioactive peptide *Carcinus maenas*	4E‐33
Crustacean hyperglycemic hormone	ABQ41269	ES.k41.R3771434	691	5E‐24	ACN87216	Crustacean hyperglycemic hormone precursor *Charybdis japonica*	8E‐27
Calcitonin‐like diuretic hormone	ACX46386	ES.c66980_g1_i1	608	1E‐47	ACX46386	Prepro‐calcitonin‐like diuretic hormone *Homarus americanus*	1E‐53
Corticotropin‐releasing factor‐like diuretic hormone	AAF54421	–	–	–	–	–	–
Ecdysis‐triggering hormone	AAF47275	–	–	–	–	–	–
Eclosion hormone	AAA29310	ES.21_comp1319_seq1	536	3E‐10	BAO00951	Eclosion hormone 2 *Nilaparvata lugens*	2E‐16
FMRFamide‐like peptide (myosuppressin)	ACX46385	ES.265178	343	2E‐34	BAG68789	Myosuppressin‐like neuropeptide precursor *Procambarus clarkii*	9E‐36
FMRFamide‐like peptide (neuropeptide F)	AEC12204	ES.23_comp4135_seq0	700	3E‐26	AEC12204	Preproneuropeptide F I *Litopenaeus vannamei*	5E‐27
FMRFamide‐like peptide (short neuropeptide F)	AAU87571	ES.k61.S126078	378	4E‐08	ETN63818	Short neuropeptide F precursor *Anopheles darlingi*	2E‐13
FMRFamide‐like peptide (sulfakinin)	ABQ95346	–	–	–	–	–	–
FMRFamide‐like peptide (other)	BAE06262	ES.63534	521	6E‐06	AAR19420	FMRFamide‐like peptide precursor *Periplaneta americana*	1E‐08
Insulin‐like peptide	AAS65048	–	–	–	–	–	–
Inotocin	ABX52000	ES.c73008_g1_i1	538	3E‐17	BAO00906	Arginine vasotocin precursor *Paralichthys olivaceus*	2E‐24
Leucokinin	AAF49731	–	–	–	–	–	–
Neuroparsin^a^	ACO11224	ES.31_comp27103_seq0	522	1E‐15	ACO10490	Neuroparsin‐A precursor *Caligus rogercresseyi*	4E‐16
Orcokinin	ACB41787	ES.k51.J2644345	765	2E‐59	Q9NL82	Orcokinin‐like peptide 4 Precursor *Procambarus clarkii*	1E‐77
Periviscerokinin/pyrokinin	NP_001104182	–	–	–	–	–	–
Proctolin	CAD30643	–	–	–	–	–	–
Red pigment concentrating hormone	ABV46765	ES.c39108_g1_i1	620	9E‐18	ABV46765	Red pigment concentrating hormone *Macrobrachium rosenbergii*	4E‐17
Adipokinetic hormone	EFX68649	–	–	–	–	–	–
RYamide	EDP28140	–	–	–	–	–	–
SIFamide	BAC55939	ES.23_comp6805_seq0	935	7E‐25	Q867W1	SIFamide precursor *Procambarus clarkii*	8E‐24
Tachykinin‐related peptide	ACB41786	ES.c73859_g1_i1	1289	6E‐37	BAD06363	Preprotachykinin B *Panulirus interruptus*	1E‐37

a Candidates for these genes also reported by Toullec et al. (2013).

Searching KrillDB for circadian‐related transcripts (core, regulatory, and clock‐controlled genes) generated largely similar results, albeit that the majority of transcripts returned from the total assembly were longer than their KrillDB counterparts (Table 6). In KrillDB, a 303 bp 3′ fragment was the only evidence found of *Es‐bmal1*, and *Es‐per* was represented by a 2,339 bp fragment, both less extensive than the results from the total assembly. Conversely, *Es‐tim* was represented by 4,047 bp contig in KrillDB and *Es‐cry1* by a 1,819 bp contig, both covering the complete coding sequence in contrast to the shorter, fragmented transcripts in the total assembly. By visual inspection of the results, no equivalent was found in KrillDB for the total assembly contigs identified as representing the genes *PP2A microtubule star, PP2A widerborst,* and *takeout*—in the case of the first two genes the top result from KrillDB was a less compelling candidate compared to the output from the total assembly and each was also found to be present in the total assembly, but rejected in favor of the contigs listed in Table 3. In the case of *takeout*, no candidate contig was found in KrillDB.

**Table 6:** Comparison of circadian‐related contigs from the total assembly of SuperbaSE and equivalents found in KrillDB. Longest accepted candidate contig shown for each assembly. “Agreement” shows percentage of identical residues in the largest HSP in each search result alignment

Gene	Total assembly contig	Length	KrillDB contig	Length	Agreement (%)
*Bmal1*	ES.21_comp39465_seq0	1,150	ESS133965	303	100
*Clock*	ES.21_comp24303_seq0	1,903	ESS034516	1,795	100
*Period*	ES.23_comp19428_seq0	4,476	ESS133963	2,339	99
*Timeless*	ES.23_comp16712_seq0	3,415	ESS040526	4,047	99
*Cryptochrome 1*	ES.23_comp21133_seq0	1,100	ESS023688	1,819	99
*Cryptochrome 2*	ES.154212	2,146	ESS118473	1,855	100
*Casein kinase IIA*	ES.21_comp3643_seq1	2,556	ESS071624	1,825	91
*Casein kinase IIB*	ES.194593	1,276	ESS051147	805	97
*Clockwork orange*	ES.21_comp15386_seq1	2,301	ESS049809	2,111	95
*Ctrip*	ES.31_comp9752_seq0	8,148	ESS002511	2,454	100
*Doubletime*	ES.25_comp4349_seq1	2,480	ESS096454	1,925	91
*Lark*	ES.k31.S5232406	1,199	ESS098132	1,058	70
*Nejire*	ES.31_comp5882_seq0	6,924	ESS048098	2,180	98
*Nemo*	k21.S7956922	2,038	ESS021045	2,238	100
*Par Domain Protein 1*	ES.k41.J3781389	1,835	ESS017186	465	75
*Pigment‐dispersing hormone*	ES.k51.S2746663	387	ESS069399	362	93
*PDH receptor*	ES.21_comp12084_seq0	1,588	ESS048516	1,557	100
*Protein phosphatase 1*	ES.k51.J2636217	1,870	ESS073678	2,246	99
*PP2A ‐ Microtubule star*	ES.23_comp1445_seq0	2,263	ESS030735^a^	1,334	58
*PP2A ‐ Twins*	ES.21_comp997_seq2	3,375	ESS126945	2,979	100
*PP2A ‐ Widerborst*	ES.104836	4,190	ESS023456^a^	3,762	72
*Rev‐erb/E75*	ES.25_comp2884_seq0	3,602	ESS094384	3,620	99
*Shaggy*	ES.27_comp3209_seq0	2,222	ESS074789	1,350	96
*Supernumerary limbs*	ES.25_comp34876_seq0	1,843	ESS133964	1,350	100
*Takeout*	ES.21_comp3588_seq0	1,090	–	–	–
*Timeout*	ES.23_comp37221_seq0	3,067	ESS130300	2,078	99
*Vrille*	ES.25_comp4277_seq0	2,021	ESS123361	1,522	100

a These contigs were rejected as equivalent to the query contigs on visual inspection.

### 3.4 SuperbaSE: The online *Euphausia superba* transcriptome database

**Figure 8:**
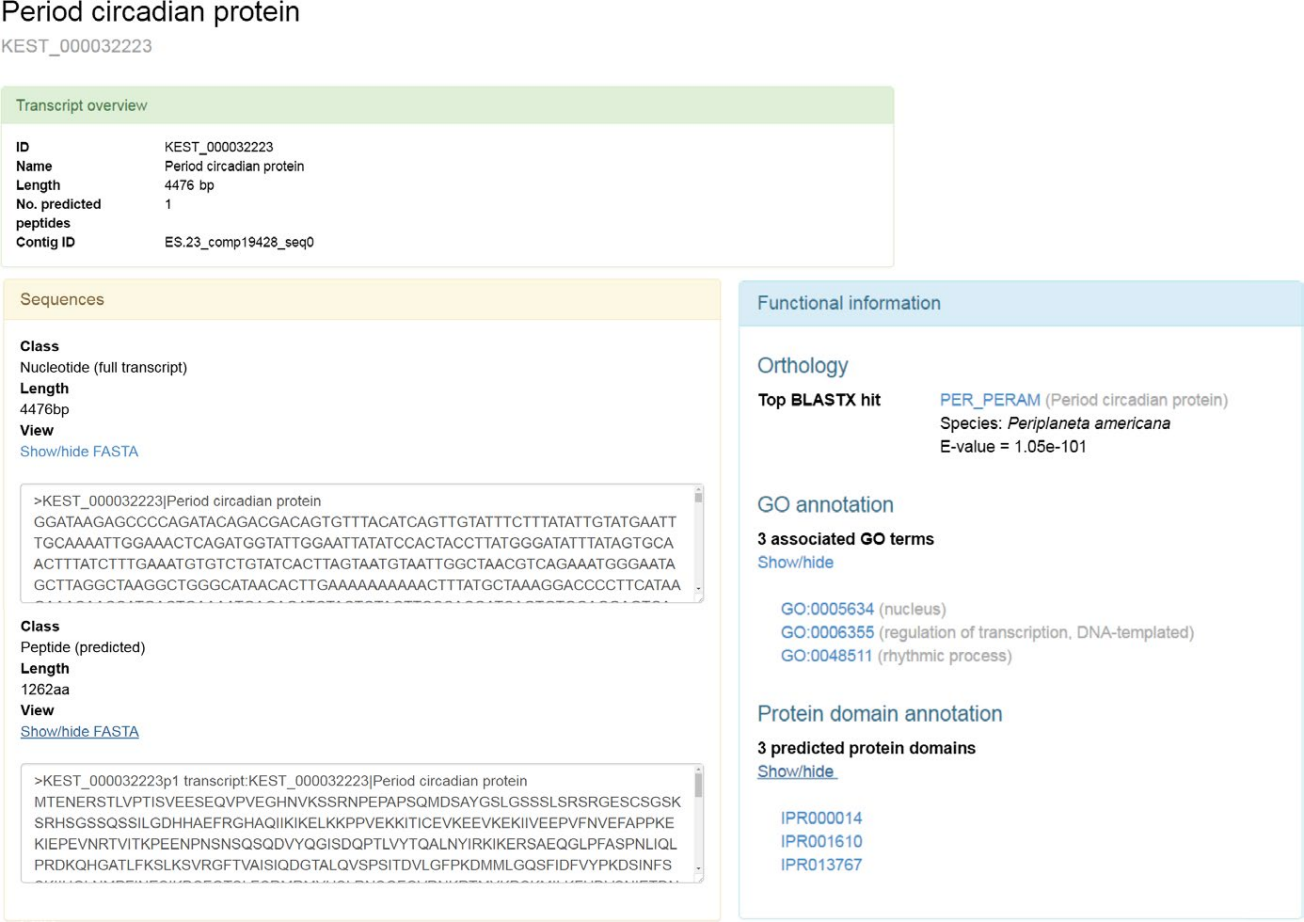
Sequence and annotation data found in SuperbaSE for circadian gene *Es‐period*

SuperbaSE is hosted at http://www.krill.le.ac.uk. The site features a Quick Search which scans transcript/contig and GO fields for the input search text and an Advanced Search function with a wider set of options. Results can be exported as a raw FASTA file of nucleotide sequences, or individual results can be viewed on their own page further showing the predicted peptide sequence and hyperlinked annotation data that permits the retrieval of details from UniProt and other sources. Figure 8 shows the data for the circadian gene *Es‐period* as it is found on the website, including functional information. As an extra resource, the peptide assembly, coding assembly, and total assembly are available as searchable BLAST databases using the SequenceServer front end (Priyam et al., 2015), permitting fast, simple identification of genes of interest.

## 4 DISCUSSION

### 4.1 Assembly and assessment

SuperbaSE was created as a tool for gene discovery. As such, the pipeline adopted for assembly focused on generating a comprehensive set of correct and complete transcripts, and one of the major factors affecting the reconstruction of transcripts from short read data is the *k*‐mer length employed in assembly. Typically, as *k‐*mer length increases, so too does average contig length and the median coverage depth of contigs assembled, while contig number falls (Gibbons et al., 2009); low *k*‐mers therefore favor transcript diversity, while high *k*‐mers favor contiguity. Often an intermediate *k*‐mer is chosen as a compromise but from the perspective of gene discovery, in which diversity, contiguity, and accuracy are all prized, this is suboptimal. A number of studies have shown the value of a multiple *k*‐mer approach, showing that it increases the sequence information obtained for an individual transcript, increases the number of reads represented in the assembly, improves contiguity, and reconstructs transcripts not found in a single *k*‐mer assembly (Surget‐Groba & Montoya‐Burgos, 2010); that it increases the number of full‐length reconstructed transcripts (Zhao et al., 2011); and that for each *k*‐mer the resultant assembly contains unique contigs not present in others (Haznedaroglu, Reeves, Rismani‐Yazdi, & Peccia, 2012). Comparisons of different assembly packages have also shown variability in annotatable output when using the same dataset and *k*‐mer (Chang et al., 2015; Xie et al., 2014; Zhao et al., 2011). Our pipeline was inspired by such findings, employing a multi‐assembler, multi‐*k*‐mer approach to generate a large number of assemblies that were subsequently merged. We then used TransRate as a method of identifying the best‐assembled contigs among those in the merged assembly. As a test of the TransRate method, Smith‐Unna et al. (2016) performed a meta‐analysis of 155 published transcriptome assemblies in the NCBI TSA and determined that an assembly with a Transrate score of 0.22 would be superior to 50% of those assessed; while our initial assemblies each received scores of around that mark (Table 2), the total assembly consisting of the best contigs after merging and removal of duplicates was assigned a score of 0.42. Using this method increased the number of unique accessions retrieved by the coding assembly compared to various single assemblies (Supplementary Data, Table S3), despite the fact that, of these, only the coding assembly had been subject to processing to remove short, noncoding and low‐quality contigs which may otherwise have returned annotations, highlighting how information present in the read data can be missed if a comprehensive approach is not adopted.

Comparative methods of transcriptome assessment that use a related organism as a measure of completeness (O'Neil et al., 2013) focus on conserved sequence identification at the expense of novel and divergent transcripts and lose relevancy with increasing evolutionary distance between subject and reference. TransRate, in contrast, has the benefit of directly assessing contig quality based on read‐mapping metrics, identifying chimeras, gene family collapses, insertions, fragmentation, local misassemblies, and redundancy. This latter issue was further addressed through the use of BBMap's deduphe.sh at the nucleotide level and CD‐HIT at the peptide level to remove absolute duplicates. Despite these efforts, redundancy is still an issue. The total assembly contains nearly half a million contigs yet no individual assembly produced more than 160,000 contigs (data not shown). *De novo* assembly is a sensitive, error‐prone process and small differences in contig output across assemblers or *k*‐mers may produce many variants representing the same in vivo transcript. This is indicated in the number of accessions assigned to more than one contig, with those assigned to multiple contigs representing 55% of the total number. As illustrated by the example identified in the Results, that of the pro‐resilin precursor of *Drosophila melanogaster,* this can stretch to over a thousand contigs, although this particular annotation also appeared 274 times in a single assembly. This problem could be assuaged by relaxing the criteria for duplicates when running dedupe.sh and CD‐HIT ‐ by removing duplicates at 95% identity rather than 100%, for example—but again, given the focus on gene discovery, we preferred to tolerate redundancy over the possibility that correct transcripts may be lost, and we therefore offer this resource with the caveat that searches are likely to return multiple results worthy of investigation and that do not necessarily represent isoforms.

### 4.2 Functional and comparative analysis

The coding assembly of SuperbaSE compares favorably with the majority of existing molecular resources for *E. superba* in terms of the number of assembled contigs, annotated contigs, largest contig, average contig size, and N50 metric (Table 7). The recently published resource KrillDB (Sales et al., 2017) combines fragments from multiple adult libraries (Meyer et al., 2015) with Illumina sequencing of RNA libraries from krill larvae under varying CO_2_ levels and has similar metrics, including the number of predicted complete full‐length transcripts (27,928 for KrillDB, 27,413 for SuperbaSE). Both transcriptomes have a similar distribution of functional categories (Figure 1) that is not notably lacking in representation in comparison with the *D. pulex* genome.

**Table 7:** Comparison of published *E. superba de novo* assemblies. “No. of reads” shows raw read count of new sequencing based on the samples described in this table, either as reported or obtained from NCBI SRA

	Clark et al. (2011)	De Pittà et al. (2013)	Martins et al. (2015)	Meyer et al. (2015)	Sales et al. (2017)	SuperbaSE (coding assembly)
Sampling vicinity	South Orkney Islands	Ross Sea	Antarctic Peninsula	East Antarctica/Lazarev Sea	Indian Ocean	NW of South Georgia
Tissue type	Adult, whole	Adult, head, abdomen, thoracopods	Adult, head and eye stalks	Adult, whole/head, aquarium/wild	Larval, whole, aquarium‐bred	Adult, head
Experimental conditions/library type	Pooled time series	Pooled time series	Control/food deprivation/UV‐B stress	Pooled seasonal samples	Control/1000/2000 μatm pCO_2_	Pooled time series
Sequencing technology	GS FLX Titanium (454)	GS FLX Titanium (454)	GS FLX Titanium (454)	GS FLX Titanium (454)	Illumina Genome Analyzer IIx	Illumina HiSeq 2000
No. of reads	943,817	96,803	377,442	1,771,572	368,287,158	69,837,314
No. of reads used in assembly	699,248	711,148	306,182	2,608,911	33,175,931	69,837,314
No. of assembled contigs	22,177	32,217	26,415	58,581	133,962	147,450
Smallest contig	137	300	–	300	200	200
Largest contig	8,515	8,558	–	11,127	14,396	34,468
Average contig size	492	890	593	691	964	1,121
N50	–	–	666	716	1,294	1,518
No. of reported BLAST annotations	5,563	11,230	10,501	15,347	90,121	92,201
BLAST annotation source	GenBank nonredundant	NCBI nonredundant, UniProtKB	NCBI nonredundant (arthropoda)	NCBI nucleotide, UniProtKB	NCBI nucleotide, UniProtKB/TREMBL	SwissProt, UniProt arthropoda
Notes	–	Final assembly includes publicly available ESTs and reads from Clark et al. (2011).	–	Final assembly includes reads from Clark et al. (2011) and De Pittà et al. (2013)	Reads were subject to digital normalization before assembly. Final assembly includes fragments from Meyer et al. (2015)	–

Despite the broad overlap, SuperbaSE and KrillDB have some clear differences. The majority of KrillDB transcripts come from larval stage RNA extracted under experimental conditions–81% of assembled transcripts are from the larval sequencing project that this resource centers around. The other 19% come from previous adult transcriptomes assembled from a smaller number of reads generated through 454 pyrosequencing. SuperbaSE, however, derives entirely from wild‐caught adult head tissue, and it is clear that numerous transcripts are to be found in this resource and not in KrillDB and *vice versa*, with 23.6% of SuperbaSE contigs finding no match in KrillDB and 14.4% of KrillDB contigs having no match in SuperbaSE, based on a reciprocal blastn search using an E‐value threshold of 1.0e^−20^. Certain core circadian genes (*Es‐bmal1, Es‐per*) are less extensively reconstructed in KrillDB, while others (*Es‐tim, Es‐cry1*) were more fragmented in SuperbaSE. Both resources provide open access to their annotated datasets and it is apparent that they are complementary, serving to broaden the molecular information openly available for this species.

### 4.3 Circadian gene discovery

Using SuperbaSE, we have successfully identified the complete sequences for *Es‐per* and *Es‐tim*, two core circadian genes over 3.5 kb in length with low expression levels (Supplementary Data, Table S4). Previously cloned circadian genes of similarly low expression levels were well represented by extensive fragments, and deviations from the sequences obtained by high fidelity PCR were minimal with the exception of *Es‐clk*. We suggest that the causes of this particular exception are 1) a misassembly at the 3′ end of the contig ES.21_comp24303_seq0, as contig ES.31_comp16417_seq0 is in agreement with the PCR sequence, and 2) the inherent difficulty in reassembling a region showing a high level of repeats. Much of the disagreement is seen in or near an extraordinary long poly‐Q region, a common feature of *Clock* genes (Johnsen et al., 2007; O'Malley & Banks, 2008; Saleem, Anand, Jain, & Brahmachari, 2001); to our knowledge, the poly‐Q of Es‐CLK is the longest example yet found, surpassing that of the river prawn *Macrobrachium rosenbergii* (Yang, Dai, Yang, & Yang, 2006). Our findings show that *E. superba* possesses a full complement of core clock proteins in contrast to *D. melanogaster*, which lacks a CRY2, and *M. musculus*, which lacks TIMELESS and CRY1 (Reppert & Weaver, 2000). The krill clock shares this characteristic with a number of other arthropod species including the monarch butterfly *Danaus plexippus* (Zhu et al., 2008), and it has been suggested that this represents an ancestral form from which the fly and mouse clocks have been derived.

Further research is now needed to link the molecular clock to the rhythmic phenomena observed in *E. superba*. Diel vertical migration, in which krill rise to the surface at night to feed and return to deeper waters at dawn, is likely influenced by the biological clock (Gaten et al., 2008). Krill also exhibit annual rhythms with a molt‐shrink regression to a juvenile state over winter, returning to maturity in spring (Kawaguchi, Yoshida, Finley, Cramp, & Nicol, 2007), and seasonal cycling of metabolic rate (Meyer et al., 2010). At the genetic level, this may be governed by photoperiod (Seear et al., 2009), and circadian clock genes have been suggested to play a role in photoperiodic responses in *D. melanogaster* (Pegoraro, Gesto, Kyriacou, & Tauber, 2014). *Es‐cry2* has shown rhythmic expression in both light–dark cycling conditions and in constant darkness with a short circadian period of 18 hr (Teschke, Wendt, Kawaguchi, Kramer, & Meyer, 2011), a finding interpreted as evidence of a clock with a wide range of entrainment and consistent with the extreme variation in photoperiod that *E. superba* is subject to, from constant light in summer to constant darkness during winter. With a full set of core circadian genes now cloned and sequenced, this intriguing system can be further characterized, and our preliminary results from assays on the interactions of the krill clock proteins indicate that the krill clock retains the functionality of both the fly and mouse mechanisms (data will be presented elsewhere) to an extent not seen in other species so far studied.

We have furthermore identified 18 putative full coding sequences for regulatory and clock‐controlled genes plus extensive fragments for three more such genes of interest and 21 preprohormone candidate contigs, the majority not previously reported. As an example of its usage outside the field of chronobiology, SuperbaSE has also been employed in the identification of an ancient conserved noncoding element in the 5′ region of the *Not1* gene (Davies, Krusche, Tauber, & Ott, 2015).

## 5 CONCLUSION

We have described the assembly and annotation of SuperbaSE, a new transcriptome resource for *Euphausia superba*. Using this resource, we have successfully identified an extensive suite of circadian and clock‐related genes, including some that had proven difficult to clone using traditional methods. This has laid the groundwork for the molecular dissection of the circadian clock in this vital species in order to understand how these components interact to generate genetic, metabolic, physiological and behavioral rhythms, and perhaps even seasonal rhythmicity. From the total assembly, a subset of 147,450 coding contigs were identified, 92,201 of which were annotated by 23,931 unique UniProtKB accessions and then further annotated with GO terms, KEGG pathway IDs, Pfam domains, Enzyme Codes, and InterProScan identifiers. The annotated dataset and total assembly have been made freely available at SuperbaSE, a user‐friendly searchable database hosted at http://www.krill.le.ac.uk.

## CONFLICT OF INTEREST

None declared.

## AUTHOR CONTRIBUTIONS

BJH performed PCR, cloning and sequencing with contributions from PS, *de novo* transcriptome assembly and annotation, transcriptome analysis and wrote the manuscript with input from all authors. ÖÖ performed mRNA extraction, cDNA synthesis, degenerate PCR, cloning, and sequencing. NJD developed the website. EG and GT collected samples. ER, EG, CPK, and GT conceived the study.

## 6 DATA ACCESSIBILITY

Sequences of cloned circadian genes are available in GenBank under accessions KX238951, KX238952, KX238953, KX238954, and KX238955, as are the sequences of the total assembly under accession GFCS00000000. Read data were submitted to NCBI SRA under accession SRR4408478. Annotated coding and peptide assembly sequences are available at http://www.krill.le.ac.uk.
