# Supplementary material for: The Euphausia superba transcriptome database, SuperbaSE: An online, open resource for researchers
Source: Ecol Evol. 2017 Jun 28;7(16):6060–77. doi: 10.1002/ece3.3168 (PMC6077532; doi:10.1002/ece3.3168)
Supplement: Supplementary file 1 [file ECE3-7-6060-s001.docx]

**Supplemental Data**

**Molecular cloning**

The mRNAs from all wild catches (see Materials and Methods) were pooled together and then used (diluted) for first strand cDNA synthesis. To produce cDNA for degenerate PCR we used ImpromII^TM^ Reverse Transcriptase and a mixture of oligo-dT and random hexamer primers, according to the manufacturer’s instructions (Promega). For rapid amplification of complementary DNA ends (RACE), we used Superscript III RT module and either oligodT or random hexamer primers (3’ RACE or 5’ RACE) according to the GeneRacer^TM^ (Thermo Fisher Scientific, UK) protocol.

To identify *E. superba bmal1, clock,* and *cry1* we performed degenerate PCR on the whole head cDNA template described above. For *Es-bmal1* and *Es-clock* we aligned orthologous sequences from other species to identify conserved regions suitable for designing degenerate primers (Table S1). For *Es-cry1* we used sequences from Merlin *et al.*, (2006). To clone *Es-cry2* we used primers based on the sequence described by Mazzotta *et al.*, (2010). To identify *E. superba* *period* and *timeless* we performed BLAST searches of the Sequence Read Archive (NCBI) restricted to dataset SRX026165, at the time the only transcriptomic resource available for *E. superba* (Clark *et al.*, 2011). This dataset was queried using the tblastn function with the coding sequences of *Drosophila melanogaster period* (NP_525056) and *timeless* (NP_722914), *Daphnia pulex period* (EFX76293) and *timeless* (EFX87311) and *Gryllus bimaculatus timeless* (BAJ16356). For any hits with an alignment score greater than 50 the contig was retrieved from the archive and stripped of adapter sequences.

Sequences obtained from these methods were translated using ExPASy Translate (<http://web.expasy.org/translate/>) and the derived peptide sequences used to search the NCBI non-redundant (NR) protein database to ascertain identity. Those deemed likely to represent a fragment of an *E. superba* ortholog of the gene of interest were subsequently subject to 5’ and 3’ RACE extension.

The GeneRacer^TM^ kit was used to extend fragments to obtain the full coding sequence. Both 5’ and 3’ RACE and nested RACE was conducted per the manufacturer’s instructions with the exception that equal volumes of GeneRacer primers and gene-specific primers were used (1 µl each). RACE-amplified fragments were purified, cloned and sequenced as above. *Es-timeout* is an ortholog of *D. melanogaster timeout* (mammalian *Timeless)*. Evidence for its role in the circadian clock is limited and so cloning and sequencing of the full sequence was not conducted. However RACE extension was used to bridge the gaps between fragments identified in the head transcriptome.

Degenerate PCR was performed using KAPA Taq DNA Polymerase (Kapa Biosystems), RACE PCR was performed using Platinum Taq DNA Polymerase High Fidelity (Thermo Fisher Scientific, UK), and coding sequence confirmation PCR was performed using Q5 High Fidelity DNA Polymerase (NEB) or, for *Es-cry1* and *Es-cry2,* Expand High Fidelity PCR System (Roche). Sequences amplified in these reactions were gel purified using QIAquick Gel Extraction kit (Qiagen), cloned into pCR®4-TOPO® (Thermo Fisher Scientific, UK) or pMiniT (NEB) vectors and sequenced by the Sanger method (PNACL, University of Leicester and GATC Biotech). All primers and thermal profiles used in these reactions are shown in Table S2 and Figure S1.

***De novo* transcriptome assembly**

The following are examples of commands used at each stage. Figure S2 shows the pipeline for the creation of the *E. superba* head transcriptome.

Trimmomatic:

trimmomatic PE -threads 16 rawreads1.fastq rawreads2.fastq readfile1.fastq readfile1_unpair.fastq readfile2.fastq readfile2_unpair.fastq AVGQUAL:20 LEADING:3 TRAILING:3

Assembly:

Bridger.pl --seqType fq --left readfile1.fq --right readfile2.fq -k 25 --CPU 16 --out /outputpath/outputfolder/ --clean

Trinity --seqType fq --JM 400G --left readfile1.fq --right readfile2.fq --output /outputpath/outputfolder/ --CPU 16 --min_contig_length 200 --bflyCPU 4

transabyss --pe readfile1.fq readfile2.fq --outdir /outputpath/outputfolder/ --name transabyss21 --length 200 --threads 16 -k 25

SOAPdenovo-Trans-127mer all -s euphausia.config -o /outputpath/outputfolder/ -R -K 25 -L 200 -p 16

Merging assemblies using Trans-ABySS:

transabyss-merge assembly21.fasta assembly31.fasta assembly41.fasta assembly51.fasta assembly61.fasta assembly71.fasta assembly81.fasta assembly91.fasta --mink 21 --maxk 91 --prefixes k21. k31. k41. k51. k61. k71. k81. k91. --out transabyss.merge.fasta

Renaming contigs for subsequent merger using the cat command

sed -i 's/>/>k71./g' assembly71.fasta

cat *.fasta > merged.fasta

Removing duplicates at the nucleotide level:

dedupe.sh in=merged.fasta out=merged_dedupe.fasta outd=duplicates.fasta overwrite=t absorbrc=t

Running TransRate:

transrate --assembly merged.fasta --left readfile1.fq --right readfile2.fq --threads 8 -o /outputpath/outputfolder/

Create the peptide assembly:

perl TransDecoder.LongOrfs -t totalassembly.fasta

perl TransDecoder.Predict -t totalassembly.fasta --retain_long_orfs 300 --single_best_orf

Removing duplicates at the peptide level:

cd-hit -i transdecoder.pep -o transdecoder.cdhit.pep -c 1 -n 10 -M 60000

**Abundance estimation**

Abundance estimation was performed on the good contigs (per TransRate) of a single Bridger assembly generated with *k*-mer 25 to avoid the impact of contig redundancy, which could exacerbate issues with multi-mapping reads - although not as comprehensive in terms of transcript reconstruction as the output of the multi-assembler, multi-*k*-mer approach, the majority of genes will be represented in some form in a single assembly. RSEM (Li and Dewey, 2011) was selected as the estimation method using Bowtie alignment via the Trinity plug-in *align_and_estimate_abundance.pl* to generate transcripts per million (TPM) abundance values. Contigs in this assembly were BLAST annotated using the already annotated coding assembly, permitting the identification of fragments in the single assembly representing the genes of interest. Abundance data for circadian genes is shown in Table S4.

Table S1: Accessions of sequences used to identify conserved regions in BMAL1 and CLOCK for the purposes of designing degenerate PCR primers.

| **Protein** | **Species** | **Accession** |
| --- | --- | --- |
| CLOCK | *Mus musculus* | AAC53200 |
|  | *Gallus gallus* | NP_989505 |
|  | *Xenopus laevis* | NP_001083854 |
|  | *Danio rerio* | NP_571032 |
|  | *Drosophila melanogaster* | NP_523964 |
|  | *Thermobia domestica* | BAJ16353 |
|  | *Antheraea pernyi* | AAR14936 |
|  | *Macrobrachium rosenbergii* | AAX44045 |
| CYCLE/BMAL1 | *Mus musculus* | NP_031515 |
|  | *Gallus gallus* | AAL98706 |
|  | *Xenopus laevis* | AAW80970 |
|  | *Danio rerio* | AAF64394 |
|  | *Drosophila melanogaster* | NP_524168 |
|  | *Antheraea pernyi* | AAR14937 |
|  | *Thermobia domestica* | BAJ16354 |

Table S2: Primers used to clone the circadian genes of *E. superba*.

| Gene | Use | Forward | Reverse | Thermal profile |
| --- | --- | --- | --- | --- |
| *Es-bmal1* | Degenerate PCR | GICGDMGIGAYAARATGAA | TTYIRCRTCDATIARIAKYTT | 1 |
|  | Degenerate PCR nested | TIACBGTIYTIMGVATGGC | GAIARYTGYTCYTTIACYTT | 2 |
|  | Semi-degenerate PCR | TIACBGTIYTIMGVATGGC | GGATGAAGGATGTCAAACCATGATGTCC | 13 |
|  | RACE 5' phase 1 |  | TCCATCGGCAGCCTGCAATATCAAG | 3 |
|  | RACE 5' phase 1 nested |  | ACGCAGCACTGTGAGCTTGTCTAAC | 5 |
|  | RACE 3' phase 1 | CCTACATTATTCACAGAGTGAGCTTCTT |  | 4 |
|  | RACE 3' phase 1 nested | GGACATCATGGTTTGACATCCTTCATC |  | 6 |
|  | RACE 3' phase 2 | GCGACTGCAAAGTACCTGGCGAAC |  | 11 |
|  | RACE 3' phase 2 nested | CTGCAAAGTACCTGGCGAACATTCA |  | 12 |
|  | Full coding sequence | CATTCAATCATGTTCGGTCTGG | CAGTCTTCGCAGATTATGGCA | 18 |
| *Es-clock* | Degenerate PCR | GARAAGAARMGWMGAGATCA | TCWGGYYTBGAATTSMACTG | 1 |
|  | Degenerate PCR nested | GARATWCRACAGGAYTGGAA | GTYTGNARCCADATCCA | 2 |
|  | RACE 5' phase 1 |  | GTGCGTGAACTCCTCATTGGAGAGG | 7 |
|  | RACE 5' phase 1 nested |  | TCATTGGAGAGGAAGGAGGGTTTCCA | 9 |
|  | RACE 3' phase 1 | CTGGAAAGGGTACATCATGTTACTATC |  | 8 |
|  | RACE 3' phase 1 nested | GGTACATCATGTTACTATCGCTTTCT |  | 10 |
|  | RACE 3' phase 2 | GGCTAACAGCAGCTCTCATAATCTTG |  | 11 |
|  | RACE 3' phase 2 nested | CAGCAGCTCTCATAATCTTGAAGACA |  | 12 |
|  | RACE 3' phase 3 | GTAGGACCAAGCCTGACCATGATC |  | 11 |
|  | RACE 3' phase 3 nested | CAGAGAGTGAGAGTGACCGGGATCT |  | 12 |
|  | Full coding sequence | ATGCCATTACATGAATTCCTGAGG | GTGCATCTTTCACTTCTTGGGT | 18 |
| *Es-cry1* | Degenerate PCR ^a^ | TTCGATGGAGAGASTGCAGGTACYAA | ACCCACATCCARTTICC | 19 |
|  | RACE 5' phase 1 |  | ACCTTGGAAGCAATAGAGCTGACTTT | 3 |
|  | RACE 5' phase 1 nested |  | CAGATCTTCAAGACTTTCCGAGAGGAA | 5 |
|  | RACE 3' phase 1 | CGTACGGCTGTGTCCTGCTTTCTAA |  | 4 |
|  | RACE 3' phase 1 nested | CAGTGAGTGCAGGCAACTGGATGT |  | 6 |
|  | Full coding sequence | AGAGTCTACAATGACCAACACTGG | GGTCATATTTATGTTCCTTAAAGCTTCATTC | 20 |
| *Es-cry2* | Full coding sequence ^b^ | TGTGCACAAGAACTTGCTTA | CTCTGTTCTCTGAGGCCTTATT | 21 |
| *Es-period* | Fragment PCR | CCATATTTGGGTCATTTACCTCAG | CTTCTGGTCCCTTTATTACTCTATGT | 14 |
|  | RACE 5' phase 1 |  | CAATAGTGGTAGATCTTCAGGATGGTAG | 15 |
|  | RACE 5' phase 1 nested |  | CACAGAGTTACCGGTGAGGTCCTGA | 10 |
|  | RACE 3' phase 1 | TGGCTGTGCTTTGTGAATCCATGGAC |  | 16 |
|  | RACE 3' phase 1 nested | GGCCAACATAGAGTAATAAAGGGACCAG |  | 17 |
|  | RACE 3' phase 2 | GGAACCTGCAGAGAAAAGACGACGT |  | 16 |
|  | RACE 3' phase 2 nested | AGACGACGTACCTTAGCACGAATGAT |  | 17 |
|  | RACE 3' phase 3 | GTCCAGGATCCTACCGTCATGTTCA |  | 16 |
|  | RACE 3' phase 3 nested | CAGAAGCAGCGGACTCATAAACATTC |  | 17 |
|  | Full coding sequence | GGCTAAGGCTGGGCATAAC | ACTGTGACTTCAATGGGTTGTG | 18 |
| *Es-timeless* | Fragment PCR | GAACCTCTTTGCTCAACGCTTTG | GAGACTGGATCCGACGTTGATATA | 14 |
|  | RACE 5' phase 1 |  | TCTCTTGCTTCTGCTCTCGGTACATC | 15 |
|  | RACE 5' phase 1 nested |  | CTCTCGGTACATCAAGGAAATCAGCTG | 10 |
|  | RACE 3' phase 1 | TCCTTGATGTACCGAGAGCAGAAGC |  | 16 |
|  | RACE 3' phase 1 nested | GATGTACCGAGAGCAGAAGCAAGAG |  | 17 |
|  | RACE 3' phase 2 | GAAGAAGTGCGATCATGCGGAGTCA |  | 16 |
|  | RACE 3' phase 2 nested | GGAGGCACTGAAGGAAGTTCTCTTC |  | 17 |
|  | Full coding sequence | GGTGGAAGAGCCGCTACATA | GGTATGACCTTGACTTCAGCT | 18 |
| *Es-timeout* | Fragment PCR | CAAGGTGATGCAAAATGTC | CATCCTCATCTGCAATCTGG | 18 |
|  | RACE 3' phase 1 | CCAACACAGGAAAGGCTCTCTACTC |  | 16 |
|  | RACE 3' phase 1 nested | CAGATGAAGAGAATATTGACCCAGATG |  | 17 |
|  | RACE 3' phase 1 nested 2 | GAGAATATTGACCCAGATGACAAGGA |  | 17 |

Note: Thermal profile 18 was used for a number of reactions with only the annealing temperature changed. This was as follows: *Es-bmal1* 65°C, *Es-clock* 66°C, *Es-period* 66°C, *Es-timeless* 65°C, *Es-timeout* 63°C. ^a^ Merlin *et al.*, (2006). ^b^ Mazzotta *et al.*, (2010).

Table S3: Comparison of SwissProt annotation success between individual *de novo* assemblies and coding assembly. Individual assemblies generated using different software packages and different *k*-mer values. Numbers after each assembler name indicated *k*-mer value used. Trinity forces use of a *k*-mer of 25. ‘Annotations found in no other assembly’ refers only to those assemblies listed in the table.

| Metric | Bridger 25 | Trinity | Bridger 31 | SOAPdenovo-Trans 31 | Trans-ABySS 31 | Coding Assembly |
| --- | --- | --- | --- | --- | --- | --- |
| No. of contigs | 138,067 | 157,274 | 138,362 | 96,323 | 139,444 | 147,450 |
| No. of annotated contigs | 28,584 | 30,819 | 27,904 | 21,352 | 31,360 | 67,206 |
| No. of unique accessions | 12,616 | 14,553 | 12,717 | 12,960 | 14,415 | 15,441 |
| No. of unique accessions found in no other assembly | 420 | 1,473 | 444 | 1,201 | 1,664 | 1,485 |

Table S4: Abundance estimation in transcripts per million (TPM) for *E. superba* core and regulatory clock genes.

| Circadian gene | Contig ID | TPM |
| --- | --- | --- |
| *Es-bmal1* | ES.comp69792_seq0 | 1.84 |
|  | ES.comp85796_seq0 | 2.43 |
|  | ES.comp53867_seq0 | 2.53 |
|  | ES.comp62946_seq0 | 3.68 |
|  | ES.comp42461_seq0 | 4.52 |
| *Es-clk* | ES.comp32596_seq1 | 0.77 |
|  | ES.comp32596_seq0 | 3.38 |
|  | ES.comp26173_seq0 | 4.51 |
|  | ES.comp31442_seq0 | 5.28 |
|  | ES.comp15945_seq0 | 7.63 |
| *Es-cry1* | ES.comp63811_seq0 | 1.84 |
|  | ES.comp21135_seq0 | 4.04 |
|  | ES.comp34358_seq0 | 4.64 |
| *Es-cry2* | ES.comp5516_seq0 | 21.34 |
| *Es-per* | ES.comp20192_seq0 | 3.98 |
| *Es-tim* | ES.comp28718_seq0 | 3.23 |
|  | ES.comp16463_seq0 | 5.08 |
|  | ES.comp17408_seq0 | 6.29 |
| *Es-ctrip* | ES.comp9456_seq1 | 10.31 |
| *Es-ckIIα* | ES.comp3462_seq2 | 20.47 |
| *Es-ckIIβ* | ES.comp1878_seq0 | 106.68 |
| *Es-cwo* | ES.comp16115_seq1 | 2.03 |
|  | ES.comp21158_seq0 | 4.95 |
| *Es-dbt* | ES.comp4349_seq1 | 5.25 |
| *Es-e75* | ES.comp2884_seq0 | 48.94 |
| *Es-lark* | ES.comp21231_seq0 | 4.64 |
| *Es-mts* | ES.comp1421_seq0 | 87.16 |
| *Es-nej* | ES.comp3122_seq0 | 9.69 |
| *Es-pdh* | ES.comp1987_seq0 | 64.85 |
| *Es-pdhr* | ES.comp12996_seq0 | 7.33 |
| *Es-pdp1* | ES.comp39942_seq0 | 2.58 |
| *Es-pp1A* | ES.comp1492_seq1 | 84.38 |
| *Es-sgg* | ES.comp1237_seq1 | 49.74 |
| *Es-slimb* | ES.comp34876_seq0 | 2.8 |
| *Es-takeout* | ES.comp3527_seq0 | 61.79 |
| *Es-timeout* | ES.comp37177_seq0 | 2.56 |
|  | ES.comp19904_seq0 | 4.58 |
| *Es-tws* | ES.comp2399_seq0 | 49.54 |
| *Es-vri* | ES.comp4277_seq0 | 35.76 |
| *Es-wbt* | ES.comp5250_seq3 | 9.75 |

Figure S1: Thermal profiles used to clone the core circadian genes of *E. superba.*

Figure S2: Pipeline detailing the creation of the *E. superba* head transcriptome through a multi-assembler, multi-k-mer strategy.

**Supplemental References**

Clark MS, Thorne MAS, Toullec JY, Meng Y, Guan LL, Peck LS, Moore S (2011) Antarctic krill 454 pyrosequencing reveals chaperone and stress transcriptome. *PLoS ONE*. **6**(1), 1–17.

Mazzotta GM, De Pittà C, Benna C, Tosatto SCE, Lanfranchi G, Bertolucci C, Costa R (2010) A cry from the krill. *Chronobiology international*. **27**(3), 425–445.

Merlin C, Francois M-C, Queguiner I, Maibeche-Coisne M, Jacquin-Joly E (2006) Evidence for a putative antennal clock in *Mamestra brassicae*: Molecular cloning and characterization of two clock genes -*period* and *cryptochrome*- in antennae. *Insect Molecular Biology*. **15**(2), 137–145.
